# Impairments of Synaptic Plasticity Induction Threshold and Network Oscillatory Activity in the Hippocampus Underlie Memory Deficits in a Non-Transgenic Mouse Model of Amyloidosis

**DOI:** 10.3390/biology9070175

**Published:** 2020-07-20

**Authors:** Jennifer Mayordomo-Cava, Guillermo Iborra-Lázaro, Souhail Djebari, Sara Temprano-Carazo, Irene Sánchez-Rodríguez, Danko Jeremic, Agnès Gruart, José María Delgado-García, Lydia Jiménez-Díaz, Juan D. Navarro-López

**Affiliations:** 1 Neurophysiology and Behavioral Lab, Centro Regional de Investigaciones Biomédicas, School of Medicine of Ciudad Real, University of Castilla-La Mancha, 13071 Ciudad Real, Spain; Jennifer.mayordomo@gmail.com (J.M.-C.); Guillermo.Iborra@uclm.es (G.I.-L.); Souhail.Djebari@uclm.es (S.D.); Sara.Temprano@uclm.es (S.T.-C.); Irene.Sanchez@uclm.es (I.S.-R.); Danko.Jeremic@uclm.es (D.J.); 2 Division of Neurosciences, Pablo de Olavide University, 41013 Seville, Spain; agrumas@upo.es (A.G.); jmdelgar@upo.es (J.M.D.-G.)

**Keywords:** Alzheimer model, amyloid-*β*, hippocampus, in vivo, synaptic plasticity, metaplasticity, oscillatory activity, electrophysiology, behavior, novel object recognition, habituation, LTP, LTD

## Abstract

In early Alzheimer disease (AD) models synaptic failures and upstreaming aberrant patterns of network synchronous activity result in hippocampal-dependent memory deficits. In such initial stage, soluble forms of Amyloid-*β* (A*β*) peptides have been shown to play a causal role. Among different A*β* species, A*β*_25–35_ has been identified as the biologically active fragment, as induces major neuropathological signs related to early AD stages. Consequently, it has been extensively used to acutely explore the pathophysiological events related with neuronal dysfunction induced by soluble A*β* forms. However, the synaptic mechanisms underlying its toxic effects on hippocampal-dependent memory remain unresolved. Here, in an in vivo model of amyloidosis generated by intracerebroventricular injections of A*β*_25–35_ we studied the synaptic dysfunction mechanisms underlying hippocampal cognitive deficits. At the synaptic level, long-term potentiation (LTP) of synaptic excitation and inhibition was induced in CA1 region by high frequency simulation (HFS) applied to *Schaffer* collaterals. A*β*_25–35_ was found to alter metaplastic mechanisms of plasticity, facilitating long-term depression (LTD) of both types of LTP. In addition, aberrant synchronization of hippocampal network activity was found while at the behavioral level, deficits in hippocampal-dependent habituation and recognition memories emerged. Together, our results provide a substrate for synaptic disruption mechanism underlying hippocampal cognitive deficits present in A*β*_25–35_ amyloidosis model.

## 1. Introduction

Alzheimer’s disease (AD), the most prevalent neurodegenerative disorder, is characterized by progressive memory loss together with the presence of extracellular amyloid plaques, composed of amyloid-*β* (A*β*), and of intracellular neurofibrillary tangles, composed of hyperphosphorylated tau [1]. The causal role of A*β* in the disease has been widely reported in multiple in vivo and in vitro models of early amyloidosis. As in transgenic models, acute administration of soluble oligomeric A*β* peptides causes impairments in synaptic plasticity and learning and memory processes [2]. Although evidence has shown that the progressive accumulation of A*β* has a causal role in AD, it is also clear that misfolded oligomeric forms or small A*β* aggregates, and not necessarily plaque formation, are critical in the initial state of synaptic dysfunction in early AD development [3,4]. A*β* plaques seem to serve as reservoirs of toxic oligomeric fragments that could be released by exposure to biological lipids [5]. In fact, the degree of cognitive deficits observed in early AD correlates better with A*β* assayed biochemically, than with synapse loss, plaque accumulation, tangle formation, or neurodegeneration [4,6].

In AD patients, the main oligomeric forms of A*β* contain the sequences A*β*_1–40_ or A*β*_1–42_. These peptides have been widely used to create AD models in order to understand the physiopathology of the disease since they have previously been shown to induce long-lasting dysfunctional synaptic plasticity, inflammation, and learning deficits [7,8]. However, it has been proposed that A*β*_25–35_, an isoform that can be produced in AD patients by enzymatic cleavage of A*β*_1–40_ [9], constitutes the biologically active fragment of A*β* [10] and is able to form stable aggregates [11]. Consequently, one of the non-transgenic mice models of amyloidosis most widely used has been generated by intracerebroventricular (*icv.*) administration of A*β*_25–35_.

In similar way to longer A*β* peptides, A*β*_25–35_ has been shown to induce major neuropathological signs related to early stages of AD [12]. It has extensively been used to acutely explore the pathophysiological events associated with neuronal dysfunction induced by soluble A*β* forms [13,14,15,16,17] that underlie impairments in synaptic plasticity [18] and subjacent learning and memory deterioration [19,20]. Unlike other A*β* isoforms, A*β*_25–35_ does not generate ion-permeable pores [15,16,21,22] and, consequently, provides an alternative approach to specifically study the effects of the peptide on neurotransmission while avoiding that disturbance.

Although experimental evidence has demonstrated the presence of learning and memory impairments in A*β*_25–35_ model, the neurophysiological mechanisms underlying such deficits in vivo, in alert behaving animals, remain unexplored. In the present work, we demonstrate, for the first time, the synaptic and network evidence by which A*β*_25–35_ induces the main neuropathological effects of early amyloidosis. We show that *icv.* administration of A*β*_25–35_ resulted in an alteration of the metaplastic mechanisms that modulate synaptic plasticity threshold induction in the dorsal hippocampus, facilitating LTD instead of LTP. In addition, A*β*_25–35_ injections disrupted oscillatory activity in CA1 hippocampal region by the generation of an aberrant synchronization of *theta* and *gamma* frequencies, neural rhythms needed for learning and memory processes that depend on dorsal hippocampus functionality. Because of both, synaptic and network activity dysfunction, A*β*_25–35_ showed marked amnesic effects revealed as deficiencies in the formation of hippocampal-dependent memories. Therefore, our results further support A*β*_25–35_ pathophysiological relevance in vivo and confirm it as a robust model to study metaplastic modulation in hippocampal amyloidosis in relation to memory health, in parallel to other amyloid peptide species models or AD transgenic mice models.

## 2. Materials and Methods

### 2.1. Animals

**Figure 1:**
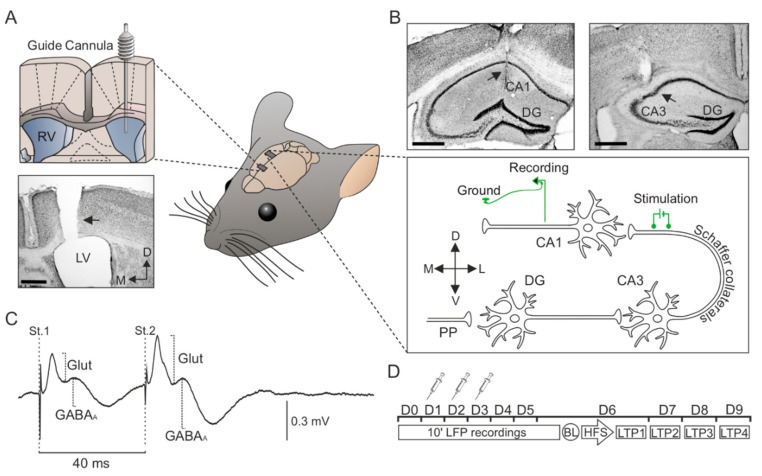
Surgery and temporal sequence of *icv.* injections, electrophysiological recordings. (**A**) Implantation of a guide cannula in the left ventricle for *icv.* vehicle or A*β*_25–35_ administration. The photomicrograph serves as histological verification of cannula position (black arrow). (**B**) Bipolar stimulating electrodes were implanted at the right *Schaffer* collateral-commissural pathway of the dorsal hippocampus, and a recording electrode was placed at the ipsilateral *stratum radiatum* underneath the CA1 area. Photomicrographs illustrate the location (black arrows) of the stimulating (right photomicrograph) and recording (left photomicrograph) electrodes. (**C**) Representation of the fPSPs evoked in the CA1 hippocampal region after paired-pulse stimulation (interval of 40 ms) of the Schaffer collaterals. The recording was obtained from a representative animal and illustrates the averaged (*n =* 50) profile of the postsynaptic response. Two different components were identified for amplitude analysis: (1) A glutamatergic fEPSP (Glut), with a latency of appearance of 2.25–4 ms after *Schaffer* collateral stimulation; and (2) a GABAergic fIPSP dependent on GABA_A_ receptors (GABA_A_), with a latency of 12–15 ms. For each component or postsynaptic potential, the maximum amplitude (peak-to-peak value) was measured for the analysis. (**D**) Vehicle or A*β*_25–35_ administration was performed on days 1–3 (D1–D3). Local field potential (LFP) recordings were collected before (D0) and after A*β*_25–35_ administration (D1-D6). From days 6 to 9 (D6–D9), the effect of A*β*_25–35_ on long-term potentiation (LTP) was checked. LTP was induced by high-frequency stimulation (HFS) of the *Schaffer* collateral pathway. The responses to paired-pulse stimulation were collected before LTP induction (Baseline; BL) and after the HFS session (LTP1-4). Scale bars, 500 μm. RV, right ventricle; LV, lateral ventricle; DG, dentate gyrus; St., stimulus; D, dorsal; M, medial; L, lateral; V, ventral; Glut, glutamate.

Experiments detailed in Figure 1 were carried out on 50 C57BL/6 male mice (24–35 g) obtained from an authorized distributor (Janvier, France). Mice were housed in group cages (*n =* 5) before surgeries, after which they were placed in individual cages and a piece of environmental enrichment for rodents was provided. They had ad libitum access to food and water throughout all experiments. All animal procedures were reviewed and approved by the Ethical Committee for Use of Laboratory Animals of the University of Castilla-La Mancha (PR-2015-01-01 and PR-2018-05-11), and followed the European Union guidelines (2010/63/EU) and Spanish regulations for the use of laboratory animals in chronic experiments (RD 53/2013 on the care of experimental animals: BOE 08/02/2013).

### 2.2. Surgery for Chronic Recording and Aβ Injection Experiments

Animals were anesthetized with 0.8–1.5% isoflurane delivered via a mouse anesthesia mask (David Kopf Instruments, Tujunga, CA, USA). The anesthetic gas was supplied from a calibrated Fluotec 5 (Fluotec-Ohmeda, Tewksbury, MA, USA) vaporizer, at a flow rate of 1–2 L/min oxygen (AstraZeneca, Madrid, Spain). Mice were implanted with bipolar electrodes aimed at the ipsilateral *Schaffer* collateral/commissural pathway of the dorsal hippocampus (Figure 1B; 1.5 mm lateral and 2 mm posterior to bregma, and 1.3 mm from the brain surface). A recording electrode was aimed at the CA1 *stratum pyramidale* (Figure 1B; 2.2 mm lateral and 1.2 mm posterior to bregma, and 1.3 mm from the brain surface). Electrodes were made from 50 mm, Teflon-coated, tungsten wire (Advent Research, Eynsham, UK). A bare silver wire was affixed to the bone as ground. All the implanted wires were soldered to a six-pin socket (RS Amidata, Madrid, Spain) and were then fixed to the skull with dental cement ([23]).

For the *icv.* administration of vehicle or A*β*_25–35_ to perfuse the dorsal hippocampus [24], the selected animals were also implanted chronically with a blunted, stainless steel, 26-G guide cannula (Plastic One, Roanoke, VA, USA) in the CA3-CA1 area, contralaterally to the hippocampal stimulating and recording electrodes (1 mm posterior to bregma, 0.46 mm lateral to midline, and 1.4 mm below the brain surface [23]). The tip of the cannula was aimed so as to be located ~0.25 mm above the infusion target. Injections were carried out with a 33-G cannula, 0.25 mm longer than the implanted guide cannula and inserted inside it (Figure 1A).

### 2.3. Electrophysiological Recordings in Behaving Animals

Recording sessions started one week after surgery. The local field potential (LFP) activity and field postsynaptic potentials (fPSPs) were recorded from alert behaving mice with Grass P511 differential amplifiers through a high-impedance probe (2  ×  1012 Ω, 10 pF). LFPs were recorded from the hippocampal CA1 area in the absence of any electrical stimulation, with the behaving animal placed in a small (5  ×  5  ×  5 cm) box before (baseline values) and after *icv.* injections during 7 consecutive days (Figure 1D). Recordings were performed for 10 min from which up to 3 min of recording, free of unwanted artifacts, was selected for spectral analysis. We selected the following frequency bands to be analyzed: low frequencies (1–40 Hz) and high frequencies (60–80 Hz). In addition, *theta* (4–12 Hz) and *gamma* (60–80 Hz) bands were specifically analyzed. The power spectrum of the hippocampal activity was computed with Spike2 software, using the fast Fourier transform with a Hanning window and expressed as mean power spectrum for each frequency band across the sessions [23,25].

For fPSPs, electrical stimuli presented to *Schaffer* collaterals consisted of 100 μs, square, biphasic pulses presented alone, paired, or in trains. fPSP baseline values were collected 15 min before induction of long-term potentiation (LTP) using paired pulses (40 ms interstimulus intervals), and the stimulus intensity was set well below the threshold for evoking a population spike, ~35% of the intensity necessary for evoking a maximum fPSP response [23,26]. An additional criterion for selecting stimulus intensity was that the second stimulus should evoke a larger (>20%) fPSP than the first. For LTP induction, animals were presented with an HFS session consisting of five 100 Hz, 100 ms trains of pulses at a rate of 1/s repeated six times, at intervals of 1 min. Evolution of fPSPs after the HFS protocol was followed for 30 min at the same stimulation rate (one stimulus every 20 s). Additional recording sessions (15 min) were carried out for three consecutive days (Figure 1D).

The different components of the fPSPs analyzed were identified according to their latency of appearance (see Figure 1C). Stimulation of the *Schaffer* collaterals evoked a glutamatergic wave with a latency of 2.25–4 ms as the main field excitatory postsynaptic potential (fEPSP) component of the postsynaptic response, closely followed by a field inhibitory postsynaptic potential (fIPSP), GABA_A_ receptor-dependent postsynaptic potential with a latency of 12–15 ms [27,28].

### 2.4. Open Field Habituation Task

The open field habituation task was carried out to determine the effect of A*β* on mice habituation learning, as previously described [24]. Briefly, mice were exposed to the open field twice, 24 h before (training trial, initial exposure) and 1 h after single *icv.* drug injection (retention trial, re-exposure; see experimental design in Figure 4A). A square open field arena with an area of 40 × 40 cm and walls 30 cm high was used. A mouse was placed in the center and was allowed to move freely for 15 min while being recorded by a video camera mounted above the open field with an actimeter AC-5 (Cibertec, Madrid, Spain). The recordings were scored later by a motion-recognition software (MUX_XYZ16L, Cibertec, Madrid, Spain) that detects and analyzes mouse movements (number of inner squares entered and number of rearing events (i.e., vertical movements)). At the end of each trial the surface of the arena was cleaned with 70% ethanol.

### 2.5. Novel Object Recognition Test

To further evaluate learning and memory capabilities in our in vivo mouse model of amyloidosis, object recognition experiments were conducted in an open-field arena (30 × 25 × 20 cm) built of polyvinyl chloride plastic, plywood, and transparent acrylic. The outside walls of the box were covered with metal plates, connected to ground, and the box was placed inside the set-up rack. Experiments were designed to test both short- and long-term memories as previously described [23]. Animals were habituated to the experimental conditions one day before test initiation (day 1). Experimental design is shown in Figure 4B. Animals received a single *icv.* injection on day 3. Experiments were performed by an observer blind to the treatment condition of the animals. Stimulus objects were made of plastic. There were several copies of each object, which were used interchangeably. The role (familiar or novel), as well as the relative position of the two stimulus objects, were counterbalanced and randomly permuted for each experimental animal. The open-field arena and the stimulus objects were cleaned thoroughly with 70% ethanol between trials to ensure the absence of olfactory cues. Exploration was defined as sniffing or touching the stimulus object with the nose and/or forepaws. Sitting on or going around the objects was not considered exploratory behavior. A video camera was positioned over the arena and the behavior of the mice was recorded using a video tracking and analysis system.

### 2.6. Rotarod Performance Test

General motor function and coordination were assessed by a rotarod apparatus (Harvard Apparatus, Holliston, MA, USA) with automatic timers and falling sensors [24]. Mice were placed on a 3 cm diameter black striated rotating rod located 20 cm above the floor (Figure 4C). First, animals were habituated to the apparatus by walking on the rod for 1 min at constant low-speed rotation. The test session was started afterwards. A single *icv.* injection was administered 1 h before the test session. A total of six trials were performed using an acceleration protocol from 4 to 40 rpm over a 5 min period. On each trial, the latency to fall from the rod was measured automatically. The apparatus was cleaned with 70% ethanol between trials.

### 2.7. Locomotion Test

Locomotor activity was determined by exposure to an open field arena consisting of a rectangular empty cage (40 × 23 cm arena). Animals underwent a single test session 1 h after *icv.* injection (Figure 4D). Mice were placed in the center of a cage and allowed to freely explore for 15 min. During this period, the travelled distance was automatically recorded by a LABORAS^®^ system (*Laboratory Animal Behavior, Observation, Registration, and Analysis System*; Metris B.V., Hoofddorp, The Netherlands) based on the detection of vibrations of the movements of each mouse [24]. Data were digitized using Metris software. The open field arena was a LABORAS^®^ cage built of Plexiglas (40 × 23 × 4 cm base and 40 × 23 × 11 cm top). The arena was cleaned with 70% ethanol between trials to remove odors.

### 2.8. Drugs

All chemicals used in this study were purchased from Bachem (Bubendorf, Switzerland) and dissolved in PBS (vehicle). To model focal A*β* pathology in the dorsal hippocampus in vivo, we used a non-transgenic model consisting of *icv.* microinjection of A*β*_25–35_ peptide that mimics the main hallmarks of different amyloidosis models of A*β* [20,29]. A*β*_25–35_ peptide was prepared as previously described [13,16,30,31]. Briefly, the peptide was dissolved to 1 mM in bidistilled water and stored in aliquots at −20 °C. Then aliquots were diluted in ACSF to required concentration and incubated for 24 h at 37 °C before experiments were performed. A*β* group received 9 nmol of A*β*_25–35_ (3 nmol/µL) in 3 µL of vehicle solution according to [20], while the control group was injected with 3 µL of the vehicle solution. The injection needle was left in place for 2–3 min before and after the injection, and volume was injected slowly at 0.5 µL/min as described elsewhere [23,24,27].

### 2.9. Histology

To verify the proper location of implanted electrodes and cannulas, at the end of the experiments mice were deeply anesthetized (sodium pentobarbital, 50 mg/kg) and perfused transcardially with saline followed by 4% paraformaldehyde in phosphate-buffered saline (PBS, 0.1 M, pH 7.4). Their brains were removed and cryoprotected with 30% sucrose in PB. Coronal sections (50 µm) were obtained with a sliding freezing microtome (Leica SM2000R, Nussloch, Germany) and stored at −20 °C in 30% glycerol and 30% ethylene glycol in PB until used. Selected sections including the implanted sites were mounted on gelatinized glass slides and stained using the Nissl technique with 0.1% toluidine blue to determine the location of stimulating and recording electrodes and/or the implanted cannula (Figure 1A,B), as previously shown [23].

### 2.10. Data Collection and Analysis

Recordings were stored digitally on a computer through an analog/digital converter (CED 1401 Plus). Data were analyzed off-line for quantification of LFPs and fPSPs, using the Spike2 (CED) program and the video capture system. Since synaptic responses did not show contamination by population spikes, the amplitude (i.e., the peak-to-peak value in mV during the rise-time period) of 15 successively evoked fPSPs was computed and stored for later analysis. These computed results were processed for statistical analysis using the figures that were prepared using SigmaPlot 12.0 package (SigmaPlot, CA, USA) and CorelDraw X8 Software.

### 2.11. Statistical Analysis

Unless otherwise indicated, data are represented as mean ± SEM. All calculations were performed using SPSS version 20 software (SPSS Inc., Chicago, IL, USA). When the distribution of the variables was normal, acquired data were analyzed with the two-tailed Student’s *t* test or the repeated measured two-way ANOVA, with time and treatment as within- and between-subjects factors respectively, and with a contrast analysis for a further study of significant differences. For repeated measures two-way ANOVA, Greenhouse Geiser correction was used and indicated in the text when sphericity was not assumed. Statistical significance was set at *p* < 0.05.

## 3. Results

### 3.1. Aβ Transforms LTP of Synaptic Excitation or Inhibition into LTD in the Hippocampal CA3-CA1 Synapse of Behaving Mice

**Figure 2:**
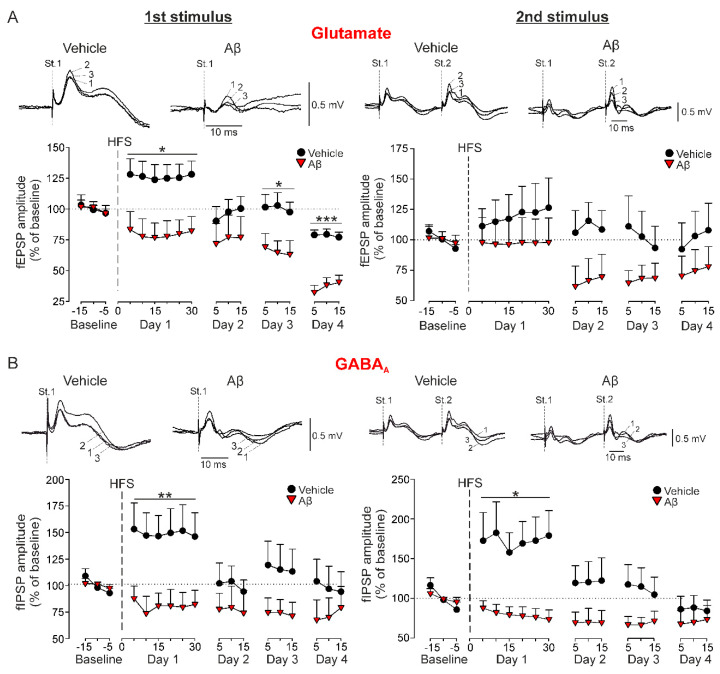
Long-term synaptic plasticity in behaving mice. The amplitude of the field postsynaptic potentials (fPSPs) evoked in vehicle and A*β*-injected mice by the first (St. 1) and second (St. 2) stimulus of paired-pulse stimulation was examined before (Baseline) and after (Day 1–Day 4) LTP induction through a HFS session. (**A**) Representative examples (averaged five times) of fEPSPs evoked before the HFS session (baseline; 1), 10 min after HFS (2) and 48 h after HFS (3) are shown above. Below, LTP of fEPSPs (mean ± SEM). * *p* < 0.05, *** *p* < 0.001 vs. vehicle. (**B**) Representative examples (averaged five times) of GABA_A_-dependent fIPSPs evoked in vehicle and A*β*-injected mice are shown above. Below, LTP of fIPSPs (mean ± SEM). * *p* < 0.05, ** *p* < 0.01 vs. vehicle.

One of the well-established hallmarks of amyloidosis models are synaptic plasticity impairments. As the functional correlate for learning and new memories formation, LTP of the glutamatergic component of the CA3-CA1 synapse has been extensively studied in amyloidosis models. However, it has not been deeply studied in the A*β*_25–35_ model. Here, to further investigate the effect of A*β*_25–35_ on CA3-CA1 synaptic plasticity in behaving animals, each animal received an HFS at *Schaffer* collaterals and evolution of the fPSPs (Figure 2) was monitored during the following 30 min and, in addition, for 15 min on the following 3 days after the HFS session. Normalization of the results was performed using day 1 baseline recordings.

As expected, LTP of the glutamatergic component was induced only in vehicle-injected control mice (*n =* 10, F_(1.56,14.04)_ = 4.125, Greenhouse-Geisser correction, *p* = 0.047) with a value of 126.2 ± 11.4% of the baseline for the first pulse (Figure 2A). However, although a LTP of the second pulse could be observed, it was not significant due to the high variability of the responses (119.1 ± 19.9%, Figure 2A, right, *p* > 0.05). On the other hand, A*β*-treated mice not only failed to show potentiation of this synaptic plasticity, but presented a progressive depression of these excitatory components that became significant on day 4 (first pulse, *n =* 8, F_(17, 119)_ = 4.8, *p* < 0.001; Figure 2A) or day 3 (second pulse, *n =* 7, F_(14, 84)_ = 2.078, *p* = 0.021; Figure 2A), reaching values of 36.9 ± 6.1% and 67.1 ± 10.7% of baseline for first and second pulse respectively.

Potentiation of synaptic inhibition was also evaluated for the IPSPs mediated by GABA_A_ activation (Figure 2B). The HFS application in vehicle-injected mice also induced a LTP of the ionotropic components evoked by the first (*n =* 9, 149 ± 18.3%, F_(2, 16.07)_ = 4.427, Greenhouse-Geisser correction, *p* = 0.029, Figure 2B) and second pulses (*n =* 8, 172.2 ± 30%, F_(8, 56)_ = 4.091, *p* < 0.001, Figure 2B), very similar to the LTP found for glutamatergic neurotransmission. In addition, depression of the fIPSP mediated by GABA_A_ activation was also induced by A*β* after the HFS protocol (first pulse, *n =* 7, 72.3 ± 10.1%, F_(8, 48)_ = 4.935, *p* < 0.001; second pulse, *n =* 7, 79.4 ± 10%, F_(8, 48)_ = 2.748, *p* = 0.014; Figure 2B). These results suggest that A*β* has a high capability for disrupting the threshold for LTP/LTD induction and consequently, upstreaming learning and memory processes that depend on hippocampal CA3-CA1 synaptic plasticity phenomena.

### 3.2. CA1 Hippocampal Oscillatory Activity Is Synchronized by Aβ in Behaving Mice

**Figure 3:**
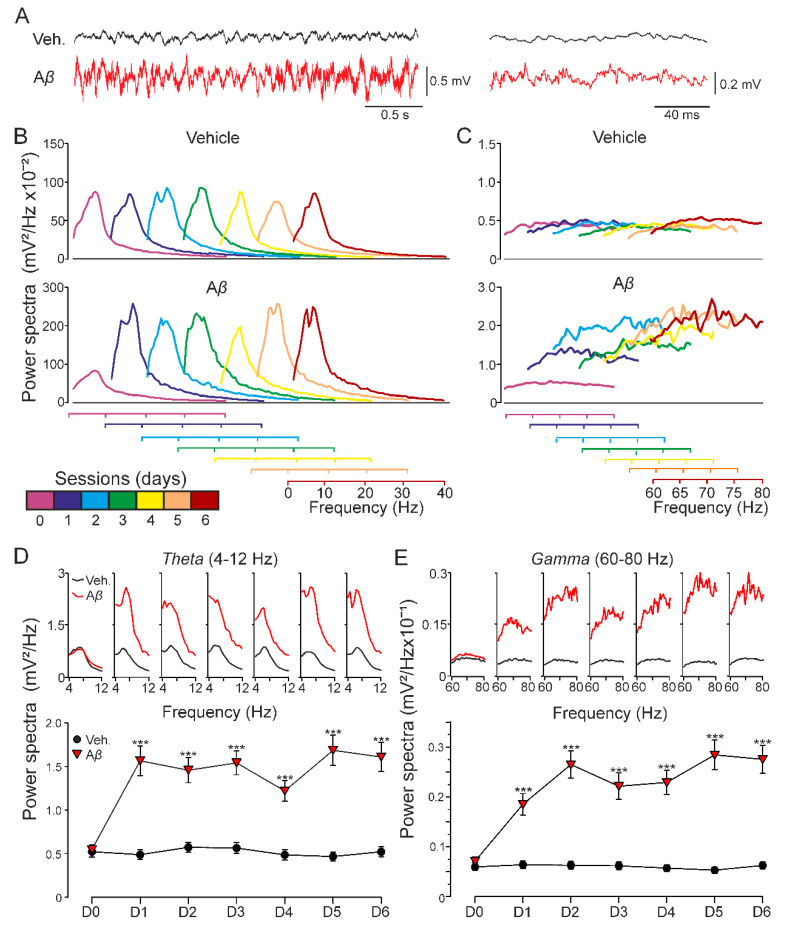
Oscillatory activity in CA1 region of behaving mice. Spectral power of 180 s LFP recordings at the CA1 area obtained on 6 consecutive days in the absence of any electrical stimulation for two experimental groups: vehicle and A*β*_25–35_. (**A**) Representative example of LFP recorded from CA1 hippocampal region in behaving mice. Note the different time scales for the traces illustrated on the left and right. (**B**) Low-frequency and (**C**) high-frequency bands were analyzed. Recordings on day 0 were collected previous to *icv.* administration (purple). Injections were performed on days 1, 2, and 3, recording 1 h after each one. LFP evolution was also followed 24, 48, and 72 h post *icv.* (days 4–6). Additionally, each day (D0-D6) spectral power of frequency intervals corresponding to hippocampal *theta* ((**D**); 4–12 Hz) and *gamma* ((**E**); 60–80 Hz) bands is represented (on top) along with the average value for that interval (below; mean ± SEM). After *icv.* administration, A*β*-injected mice showed increased spectral power at both low and high frequencies compared to vehicle-injected animals, and the same result was found for theta and gamma rhythms. *** *p* < 0.001.

As the hippocampal network activity is known to have a critical role in learning and memory processes altered in AD [32], we next examined whether our model of amyloidosis exhibited impairments in oscillatory rhythms in alert mice. As Figure 3A–C shows, A*β*-injected mice presented a sustained qualitative increase of the spectral power for low and the rest of the minor cha high frequencies that lasted at least for 6 days, indicating that A*β* is able to synchronize hippocampal oscillatory activity in a wide range of frequencies. More concretely, a quantitative spectral analysis also revealed that A*β* significantly altered *theta* (F_(2.04, 961.28)_ = 31.090, Greenhouse-Geisser correction, *p* < 0.001) and *gamma* (F_(1.58, 3000.97)_ = 75.115, Greenhouse-Geisser correction, *p* < 0.001) bands (Figure 3D,E) along the 6 days of recordings, suggesting that mechanisms needed for generation of hippocampal oscillation were damaged in our amyloidosis model.

### 3.3. Hippocampal-Dependent Memory Is Disrupted by Aβ

**Figure 4:**
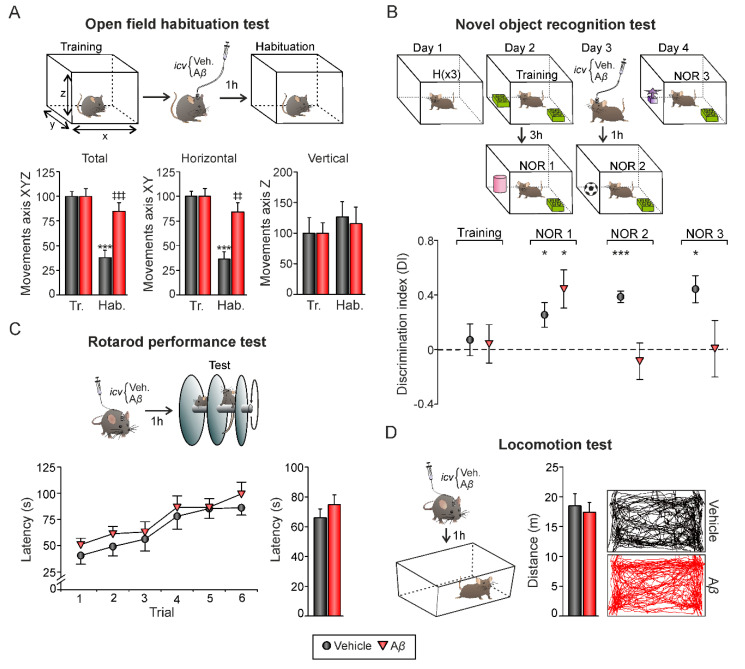
Hippocampal-dependent memory tasks. (**A**) On top, open field habituation test. Mice underwent a training session by being exposed to an open field. 24 h later, and 1 h after *icv.* administration, animals were re-exposed to the open field (habituation session). On each session, mice freely explored the environment for 15 min. Movements in the X, Y, and Z axis were detected with an infrared system. Below, total activity (movements in the XYZ axis), horizontal activity (XY axis), and vertical activity (Z axis) during training and habituation sessions (mean ± SEM) are represented. *** *p* < 0.001. Asterisks indicate differences between training and habituation sessions within each experimental group. ‡‡ *p* < 0.01, ‡‡‡ *p* < 0.001. Crosses reflect differences between A*β* and vehicle (control animals) in habituation. Note that exploratory activity decreases in the vehicle group during the habituation session compared to the training session, but also compared to the A*β* group during the habituation session. (**B**) On top, novel object recognition task. Three 5-min habituation trials were performed on Day 1. On Day 2, a 10 min training session with two identical objects occurred. A test session (NOR1) was performed 3 h later for evaluation of short-term memory, substituting one familiar object for a novel one. On days 3 and 4, NOR2 and NOR3 trials were performed to evaluate long-term memory. *Icv.* injections took place one hour before NOR2 trial. Below, discrimination index (defined as the difference in exploration time between the two objects divided by the total time spent exploring both objects) for each experimental group during trials. After *icv.* injections, only vehicle-injected mice showed positive discrimination toward the novel object. * *p* < 0.05, *** *p* < 0.001. Tr., training; Hab., habituation. (**C**) On top, Rotarod performance test. 1 h after *icv.* injections, animals performed a single session consisting of six trials. Mean latency to fall off the rod was calculated for each trial (bottom left graph) and the whole session (bottom bar plot). (**D**) Locomotion test. 1 h after *icv.* administration, mice freely explored an empty chamber for 15 min. Mean travelled distance was recorded by a LABORAS^®^ system. Right, representative movement tracking of animals from both experimental groups. No differences were found between treatments in C or D.

Our results showed that A*β*_25–35_ not only altered LTP/LTD induction mechanisms (Figure 2) but also induced an aberrant synchronization of hippocampal network activity (Figure 3) in alert mice. Both processes, synaptic plasticity, and oscillatory neural rhythms are known to support learning and memory processes that depend on hippocampus function [33]. Hence, in order to evaluate whether these impairments have a behavioral correlate, mice were challenged on two hippocampal-dependent memory tasks, the open field habituation test and the novel object recognition test (Figure 4). Additionally, the rotarod and locomotion tests were performed in order to evaluate general health state and avoid potential locomotor affectations. After *icv.* injections, all animals significantly improved their performance in the rotarod test along trials, independently from the experimental group (Figure 4C; vehicle, *n =* 9, F_(5, 40)_ = 5.494, *p* < 0.001; A*β*_25–35_, *n =* 11, F_(5, 50)_ = 7.643, *p* < 0.001). As shown in Figure 4C, no differences were found between groups when considering individual trials (*p* > 0.05 in all trials) or the whole session (F_(1, 18)_ = 0.935, *p* = 0.346), suggesting the absence of any locomotor dysfunction associated to A*β*_25–35_ administration. Consistently, the locomotion test run in an open field arena revealed similar travelled distances in A*β*- (*n =* 11; 17.41 ± 1.62 m) and vehicle-injected (control) mice (*n =* 9; 18.47 ± 2.06 m) when exposed to an empty chamber (Figure 4D; *t*(18) = 0.409, *p* = 0.687), further confirming comparable health and locomotor states. On the other hand, the open field habituation test allowed us to evaluate an elementary form of non-associative hippocampal-dependent learning [34] as the task relies upon the tendency of rodents to decrease exploratory behavior in response to repeated exposure (i.e., habituation) to a novel environment as an open field (Figure 4A). During the training session, no significant differences in exploration between mice assigned to each experimental group were found (Figure 4A; *n =* 18; XYZ axis, *t*(16) = 1.214, *p* = 0.242; XY axis, *t*(16) = 1.341, *p* = 0.199; Z axis, *t*(16) = −1.712, *p* = 0.106). 24 h later, on the retention (habituation) trial performed after the injections, only control-vehicle animals significantly decreased their exploratory movements in comparison to the training session, showing they were able to remember the open field (Figure 4A; XYZ axis, *t*(9) = 8.074, *p* < 0.001; XY axis, *t*(9) = 7.982, *p* < 0.001; Z axis, *t*(9) = −0.718, *p* = 0.491). However, the slight decrease in exploration observed in A*β*_25–35_-injected animals was not significant (Figure 4B; XYZ axis, *t*(7) = −1.848, *p* = 0.107; XY axis, *t*(7) = 1.764, *p* = 0.121; Z axis, *t*(7) = −0.416, *p* = 0.690) and evident differences in total and horizontal activity were found when compared to vehicle-injected control mice (Figure 4B; A*β* vs. veh.: XYZ axis, *t*(16) = −4.047, *p* < 0.001; XY axis, *t*(16) = −3.919, *p* = 0.0012; Z axis, *t*(16) = 0.305, *p* = 0.764), indicating hippocampal-dependent habituation memory deficits induced by A*β*_25–35_.

We then asked whether A*β* would also induce long-lasting behavioral deficits. In order to assess hippocampal-dependent memory impairments, we tested mice in the novel object recognition (NOR) test (Figure 4B). It is well established that object recognition (OR) memory formation is totally reliant on CA3–CA1 synaptic functionality in behaving mice [35]. Discrimination index was defined as the difference in exploration time between the two objects divided by the total time spent exploring both objects, for each experimental group during trials. During the training session (Day 2), all animals exhibited a discrimination index close to 0 (*p* > 0.05 for both experimental groups), indicating that mice did not present preference for any object since they spent a similar amount of time exploring both of them.

However, in NOR1 session performed 3 h later, both experimental groups showed more interest for the new object, reflected as higher DI than that observed during training (Figure 4B; vehicle (*n =* 5): DI = 0.25 ± 0.09, *t*(4) = 2.797, *p* = 0.049; A*β* (*n =* 6): DI = 0.44 ± 0.14, *t*(5) = 3.173, *p* = 0.025). 24 h later, animals were *icv.* injected and exposed to two new sessions (NOR2 and NOR3, respectively). Vehicle-injected control mice exhibited positive discrimination toward the novel object (Figure 4B; NOR2, DI = 0.39 ± 0.04, *t*(4) = 9.291, *p* < 0.001; NOR 3, DI = 0.37 ± 0.09, *t*(4) = 4.069, *p* = 0.015) whereas A*β*-injected animals explored both objects equally (Figure 4B; NOR2, DI = −0.08 ± 0.13, *t*(5) = −0.636, *p* = 0.553; NOR 3, DI = 0.007 ± 0.14, *t*(5) = 0.32, *p* = 0.976). These results confirm the behavioral correlate of the hippocampal synaptic plasticity impairments induced by A*β*.

## 4. Discussion

In the present work, the non-transgenic mice model of amyloidosis generated by *icv.* administration of A*β*_25–35_ has been neurophysiological and behaviorally characterized. Our results showed that this model displayed synaptic plasticity and oscillatory activity impairments as a consequence of compromised dorsal hippocampus functional integrity. All these disruptions were translated to hippocampal-dependent behavioral deficits, presenting this model as a valid experimental approach to study the underlying pathophysiology of early and acute hippocampal amyloidosis.

### 4.1. HFS-Induced LTP Is Transformed into LTD by Aβ_25-35_ in Behaving Mice

The dorsal hippocampus has been shown to perform primarily cognitive processes of learning and memory [36]. Such processes are supported by an increase of synaptic strength in form of long-term synaptic potentiation of different components (fEPSP and fIPSP) at the CA3-CA1 synapse (for review see [37]). Even when synaptic plasticity processes have been extensively studied with in vitro preparations, these components cannot be clearly dissected mainly because of the lack of GABAergic projections in dorsal hippocampal slices [24,38]. However, although in vivo recordings from behaving mice are technically more difficult, they allowed us to dissect and identify both CA3-CA1 synaptic components: an excitatory glutamatergic fEPSP, with a latency of appearance of 2.25–4 ms after stimulation and an inhibitory GABAergic fIPSP dependent on GABA_A_ receptors, with a latency of 12–15 ms. After a HFS protocol applied at *Schaffer* collaterals in vehicle-injected mice, we obtained the expected LTP of the glutamatergic fEPSPs recorded from CA1 [23,26,27]. This significant potentiation was more evident for the first than for the second pulse. Interestingly, post-HFS evolution of the GABA_A_-dependent fIPSPs also showed a significant potentiation, mirroring what was happening with the excitatory component. This mechanism, that cannot be observed in in vitro preparations, has recently been described in vivo [27]. Potentiation of synaptic excitation and inhibition seems to share, at least partially, the same molecular mechanisms. Activation of NMDA receptors needed for induction of glutamatergic LTP upregulates GABA_A_ receptors expression in hippocampal neurons [39,40] explaining the in vivo LTP of GABA_A_-dependent fIPSPs. In fact, it has been very recently demonstrated the importance of maintaining GABA_A_ conductance for LTP induction in the hippocampus [41]. Then, both processes, LTP of fEPSP and fIPSP would be synchronized in order to control synaptic strength, to prevent saturation and to assure the extinction of potentiation. These results suggest the precise control of the synaptic plasticity processes needed for new memories formation, with the aim of preparing the synapse for new plasticity events [27].

Our data also revealed that A*β*_25–35_ impairs LTP of synaptic excitation and inhibition at the CA3-CA1 synapse of the dorsal hippocampus. Moreover, HFS-induced LTP was transformed into LTD by A*β*_25–35_ for both components of the synaptic response. A*β*_25–35_ has been reported to block the glutamatergic LTP in rat slices and anaesthetized rats [18,42] without evidence of HFS-induced LTD. However, similar transformation has recently been described by using A*β*_1–42_ in the same synapse of hippocampal slices preparation [24]. In fact, HFS-induced LTD by A*β*_1–42_ has already been observed in vivo in the dorsal hippocampus of behaving mice [23] and in basolateral amygdaloid nucleus-insular cortex projection [43]. In all cases, LTD is observed in the glutamatergic component of the synaptic response.

These results, as well as current A*β*_25–35_ findings, might be explained by the Bienenstock, Cooper and Munro (BCM) theory of synaptic plasticity that suggests the necessity of a particular threshold for LTP induction [44]. Synaptic plasticity has been proposed to be governed by a previous activity of the same postsynaptic neuron or neural network, a process named metaplasticity [45]. Hence, metaplasticity tunes synapses and networks for plasticity processes [46]. For example, inducing metaplastic increases in the neuronal excitability by A*β*_1–42_ [23,47] or A*β*_25–35_ [16] seems to impair in the same way as the threshold for the induction of LTP and/or LTD. Moreover, we unveiled here, for the first time, the same deleterious mechanism for the impairment of the potentiation of synaptic inhibition. Potentiation of the fIPSP induced by HFS has been suggested to compensate the LTP of the glutamatergic component, in a synaptic mechanism needed for the maintenance of the excitatory/inhibitory neurotransmission balance that underlies hippocampal performance for memory formation [27,48]. It would be expected that A*β*_25–35_ would alter the GABA_A_-dependent potentiation of fIPSP as it also depends on NMDARs activation [39]. Indeed, the BCM theory proposes that the induction threshold for long-term synaptic plasticity generation is governed by slight changes in the Ca^2+^ concentration [44,49] that mainly depends on the grade of NMDARs activation. Then, higher Ca^2+^ influx are assumed to generate LTP, whereas lower changes promote LTD [50]. Thus, although HFS has been shown to induce LTP, it has also been suggested that HFS-induced LTD may occur if LTP threshold is not reached because stimulation fails to activate properly the postsynaptic neurons [44].

On the other hand, it has been shown that A*β* can facilitate the induction of LTD generated by low frequency stimulation (LFS). This LTD is mediated by both mGluRs [51,52] or NMDARs [53,54] pathways depending on the LFS protocol. However, in our experiments, different forms of synaptic plasticity were induced by a HFS protocol, which has been widely used to induce LTP [55]. Indeed, HFS can generate LTD as well. For example, corticostriatal LTD can be generated by HFS of the medial prefrontal cortex and has been shown to depend, as LTP or LFS-dependent A*β*-induced LTD, on NMDARs [56]. As we report here, HFS-induced LTD has also been obtained in basolateral amygdaloid nucleus-insular cortex projection, where A*β* causes a dopamine depletion that modifies the threshold for the induction of cortical LTP and/or LTD [43]. This process could also be linked to our results, as it has been shown that dopamine controls the threshold for LTP/LTD induction in the hippocampus [57] and hippocampal dopaminergic terminals are degenerated in amyloidopathy models [58] and AD patients [59].

Then, at the synaptic level, our results provide a new substrate to explain the effects of A*β*_25–35_ on dorsal hippocampus-dependent memory, i.e., the transformation of excitatory and inhibitory LTP synaptic response into LTD according to metaplasticity processes predicted by BCM theory of synaptic plasticity, as it has been previously described for A*β*_1–42_ [24,43]. In this manner, increases in the neural activity, such as depolarization induced by A*β*_25–35_ [16], or reduction of the membrane potential threshold for action potential firing, as induced by A*β*_25–35_ in cortical L2/3 pyramidal cells [60], might decrease the levels of response to HFS. The mechanism would shift the threshold to promote LTD, according to a model of sliding threshold for synaptic plasticity induction in response to changes in activity levels [46,61], resulting in HFS-induced LTD instead of LTP. In fact, it has been recently shown that plasticity can be restored in an amyloidosis mice model through a metaplastic mechanism via GirK channels priming in dorsal hippocampal synapses, ameliorating the synaptic plasticity and upstreaming behavioral deficits [23,24,27].

### 4.2. Aβ_25-35_-Induced Network Dysfunction and Memory Impairments

One of the main contributors to network dysfunction in amyloidosis is the early neuronal hyperexcitability [62]. A*β*-induced hyperexcitability triggers progressive epilepsy and cognitive impairments in AD [62,63]. These network dysfunctions are produced by early and subtle changes in the synaptic balance of excitation-inhibition in the hippocampus. Accordingly, slight changes in *theta* and *gamma* bands that occur in early AD have been proposed as potential predictors for the disease [64,65]. In fact, very recently it has been reported that an increase in *theta* power may be the first change in patients with dementia due to AD [66], and could be associated with enhancements in higher frequency oscillations in frontocentral regions [67]. These findings are in agreement with alterations found for *theta* and *gamma* powers as found in the present work, suggesting that A*β*_25–35_, as A*β*_1–42_ [23] or transgenic [64,68] models, also simulate these early AD hallmarks.

It is also well recognized that the non-transgenic model of amyloidosis generated by *icv.* injection of A*β*_25–35_ presents numerous and key aspects of amyloid toxicity. Deficits in hippocampal spatial memory, evaluated by using different mazes [20,69,70], or in non-spatial memory, such as spontaneous alternation or social recognition tests [71], have been described without evident locomotor activity alterations [29]. However, the physiological substrate for such hippocampal impairment has not been still provided. So, what would be the upstreaming consequences of synaptic plasticity and oscillatory activity impairments induced by A*β*_25–35_ in the dorsal hippocampus that we reported here? To answer this question, among different behavioral tests that have been well correlated with in vivo LTP recording at the CA1-CA3 synapse, open field habituation test and NOR task were chosen. Previously, the rotarod and locomotion tests allowed us to discard unpredictable effects of A*β*_25–35_ injections on locomotor function, minimizing potential biases in hippocampal-dependent behavioral tasks. The open field habituation test was selected because LTD has been proposed to play a relevant role in hippocampal-dependent learning [72], mainly associated with habituation forms of memory [73] and mechanisms to assure old memory traces extinction [33,74] and this type of synaptic plasticity has been found to be increased in amyloidosis models [51,54,75]. On the other hand, we selected the NOR test as the recognition of new objects in a previously known environment has been shown to be dependent on CA1-CA3 synapse [35] and deficits in this recognition memory have already been reported in AD patients [76] and rodents [20]. A*β*_25–35_ caused deleterious effects on both tests, suggesting that hippocampal alteration affects habituation and recognition memory formation. It is interesting to note that the same alterations have been clearly described in transgenic mouse models of AD overexpressing A*β*, such as hAPP mice [77,78] as well as non-transgenic amyloidosis models induced by A*β*_1–42_ *icv.* injections [23,24]. In these models it has also been unraveled the presence of aberrant hippocampal network activity, specifically in *theta* and *gamma* bands. Both oscillatory rhythms have been shown to be altered in amyloidosis models as a consequence of the hyperexcitability induced by A*β*_25–35_ [16,60]. Even more, interaction of both rhythms has been suggested to produce the *theta*–*gamma* coupling and facilitate memory processes [79]. Alterations in such neural code have been proposed to participate as an early electrophysiological signature of hippocampal network dysfunction in AD models [68].

## 5. Conclusions

In summary, our results have shown the presence of an A*β*_25–35_-mediated deleterious synaptic mechanism that impairs metaplasticity processes needed for memory storage. Such alteration modifies the threshold for LTP/LTD induction, impairs neural network activity, and underlies memory alterations that depend on dorsal hippocampus. These effects resemble those found in transgenic mice with amyloid overexpression or induced by other peptide species where metaplastic activation of hippocampal receptors or channels, such as ryanodine [80] or GirK [23,24] respectively, have been shown to reestablish plasticity processes. Therefore, our results represent an excellent tool to evaluate whether enabling a synaptic population for metaplasticity can restore cognitive health in amyloidosis models, that should be explored in the near future.
